# A Novel Fluorescence and SPE Adsorption Nanomaterials of Molecularly Imprinted Polymers Based on Quantum Dot-Grafted Covalent Organic Frameworks for the High Selectivity and Sensitivity Detection of Ferulic Acid

**DOI:** 10.3390/nano9020305

**Published:** 2019-02-23

**Authors:** Yu Wang, Yuzhen Wang, Huilin Liu

**Affiliations:** 1 Department of Food Science and Engineering, Shanxi Agricultural University, Jinzhong 30600, China; sxtgwy@126.com (Y.W.); wyzdzg999@163.com (Y.W.); 2 Department of Food and Chemical Engineering, Beijing Technology and Business University, Beijing 100048, China

**Keywords:** covalent organic frameworks, quantum dots, molecularly imprinted polymers, solid phase extraction, HPLC-MS, ferulic acid

## Abstract

A fluorescence and solid phase extraction (SPE) adsorption nanomaterials of molecularly imprinted polymers (MIPs) based on quantum dot-grafted covalent organic frameworks (QD-grafted COFs) was prepared by one-pot surface-imprinting synthesis method. Amino groups of silane reagent were at the surface of QDs to coordinate COFs efficiently by Schiff-base reactions, providing thermal and chemical stability to MIPs. It also reacted with the phenolic hydroxyl groups of ferulic acid (FA) through non-covalent interactions. The nanomaterials were used as fluorescence sensing and SPE adsorption toward determination of ferulic acid. The MIPs based on QD-grafted COFs had good fluorescence response ability, and quenching linearly at concentrations of ferulic acid from 0.03 to 60 mg kg^−1^, with a detection limit of 5 µg kg^−1^. At the same time, it exhibited a good SPE adsorption ability, and the FA extraction was from 1.63 to 3.11 mg kg^−1^ in grain by-products by SPE coupled with high performance liquid chromatography/mass spectrometry (HPLC/MS). The fluorescence and SPE-HPLC/MS were used for the efficient detection of ferulic acid in real samples with recovery values of 88–114% and 90–97%, respectively. Furthermore, the nanomaterials of MIPs based on QD-grafted COFs were used for FA detection with high sensitivity and selectivity, and it also increased the recycling of waste resources.

## 1. Introduction

Quantum dots are solution-processable semiconductor nanocrystals [1] with size dependent optical characteristics, probably the most attractive property of II-VI semiconductor nanocrystals [2], with the advantage of narrow emission linewidths, near-unity-photoluminance quantum yield and inherent photophysical stability [3]. The variable surface chemistry determined by capping ligands have extensively been used in the modification of quantum dots as luminescent species for imaging, detection, and biolabeling [4]. In the detection, fluorescent labeling can be a powerful tool for probing the local environment, as well as for biological imaging [5]. Many of the fluorescent labeling based on quantum dots are core-shell structures with two approaches for preparation. One example incorporates quantum dots into the core of a modified layer. Many researchers report core-shell fluorescent labeling of quantum dots capped by modified layer to enhance detection selectivity and sensitivity [6,7,8,9,10,11]. In the second case, the quantum dots are introduced to the surface of the modified layer. We have reported the introduction of the quantum dots on the surface of the molecularly imprinted polymers to improve the fluorescence quantum yield of core-shell complex [12].

In order to improve the selectivity, sensitivity, and optical performance of fluorescence labeling, porous materials have a strong interest in the detection. The covalent organic frameworks (COFs) are one of the famous porous materials linking molecular building blocks by strong covalent bonds to make crystals of extended structures [13]. Recently, COFs containing B–O, C=N, C–N, and B–N bond linkages have been constructed through thermodynamic control [14]. This directionality of covalent bonds provides a means of controlling how building units come together into predesigned structures [15], which makes COFs with highly ordered and flexible, low densities, high thermal and chemical stabilities, high porosities and surface areas, and uniform and tunable pore sizes [16,17,18]. COFs can also be used as supporting bodies for quantum dots, with an opportunity to incorporate diverse functional groups at a molecular level. However, these QD-COFs complexes are unable to directly respond to target analytes. Molecular imprinting involves polymerization in the presence of a template molecule to produce cavities in the polymer that are highly selective for that template [19,20,21]. It results in a three-dimensional polymer network wrapped around analyte molecules, and hollow structures complementary in their size, shape, and orientation of recognition sites to the analyte molecules.

In this paper, we used molecularly imprinted polymers (MIPs) based on quantum dots-grafted COFs (QD-grafted COFs) as a fluorescent probe for the high selectivity and sensitivity optosensing of ferulic acid. Here, the MIPs are intended to sensitively and selectively sense the bonding interactions between quantum dot-grafted COFs and ferulic acid, and to further transform this chemical information to detectable fluorescence signals. MIPs are a way to overcome the coexisting compounds with similar fluorescence responses to the target, which expanded the applications of optosensing methods based on quantum dots. Ferulic acid (FA), 4 hydroxyl-3-methoxy cinnamic acid, is one of the typically natural antioxidants with the free radical scavenging capability of superoxide anions, peroxyl radicals, hydroxyl and radicals [22]. Based on these properties, FA has been used as an antioxidant [23], antidotal [24], anti-inflammatory [25], and antithrombotic agent [26] in health products, medicines, and cosmetics. FA is abundant in plants and grains, including its by-products. The by-products are discarded in the processing of grain products such as grain bran, distiller’s grains, or corn silk, etc. FA extraction from rejected material is economical and practical for the recycling of waste resources. Therefore, it is important to develop a simple, effective, and low-cost method for FA extraction and determination.

## 2. Materials and Methods

### 2.1. Chemicals

The standards of ferulic acid, cinnamic acid, syringic acid, and caffeic acid were purchased from Acros Organics (Morris Plains, NJ, USA). 1,3,5-triformylphloroglucinol (TP; 99%) was purchased from Strem Chemicals, Inc., Newburypor, MA, USA. P-phenylenediamine (Pa) and mesitylene (99%) were purchased from Shanghai Macklin Biochemical Co., Ltd., Shanghai, China. CdSe/ZnS quantum dots with fluorescence excitation wavelength at 460 nm and emission centered at 605 nm were purchased from Jiayuan (Wuhan, China). Triton X-100 (99.5%), cyclohexane (99.5%), 3-aminopropyl triethoxysilane (APTES; 98%), and acetic acid (99.5%) were purchased from Sinopharm Chemical Reagent Co., Ltd., Beijing, China. Tetraethyl orthosilicate (TEOS; 98%) were purchased from J&K Scientific Ltd., Beijing, China. All reagents were at least analytical grade and purchased from Beijing Chemicals (Beijing, China). Doubly deionized water (DDW, 18.2 MΩ cm^−1^) was obtained from a Water Pro water purification system (Labconco, Kansas City, MO, USA).

### 2.2. Instrumentation

A Hitachi S-4800 scanning electron microscope (SEM, Hitachi, Tokyo, Japan) was used to observe the surface morphologies of the MIPs or non-imprinted polymers (NIPs) based on QDs-grafted COFs. The fluorescence spectra were acquired using a multifunctional microplate reader (Biotek Instruments Inc., Winooski, VT, USA). All fluorescence measurements were performed under the same conditions. The excitation wavelength was 460 nm for emission over the range of 500–700 nm. The high performance liquid chromatography (HPLC) system consisted of two LC-20AB pumps and an RF-10AXL ultraviolet detector (Shimadzu, Kyoto, Japan). A Visiprep TM-DL solid phase extraction (SPE) vacuum manifold (Supelco, Bellefonte, PA, USA) was used in the preprocessing procedure.

### 2.3. Preparation of MIPs Based on QD-Grafted COFs

COFs were synthesized using modified Schiff-base reactions by our previous paper [27]. The one-pot surface-imprinting synthesis method was used for the preparation of MIPs based on QD-grafted COFs. Triton X-100 (180 mL) and cyclohexane (750 mL) were used to prepare a reverse microemulsion system. The mixture was added to an oven-dried 25 mL round-bottom flask under vigorous magnetic stirring for 15 min. At room temperature, CdSe/ZnS QDs (1 mL), TEOS (50 μL), and aqueous ammonia (25 wt%, 100 μL) were then added to the flask. After the mixture had been stirred for 2 h, APTES (20 μL) and TpPa (0.5 mg) were added, and the mixture was stirred for another 2 h. FA (3 mg) was dissolved in ethanol solution (10 mL) and added (100 μL) to the flask. The mixture was sealed and stirred overnight. After polymerization, the product was purified by centrifugation, then washed twice with acetone and followed by double distilled water (DDW). Then the products were washed with methanol and dried by vacuum at 40 °C for 10 h.

The non-imprinted polymers (NIPs) based on QD-grafted COFs was used as a control. It was similar to the polymerization process with MIPs but without adding FA molecules.

### 2.4. Fluorescence Analysis of MIPs Based on QD-Grafted COFs

For the fluorescence experiment, the MIPs based on QD-grafted COFs (1 mg) were added into a hole of the 96-well enzyme labeling plate containing 200 μL of ethanol. After thorough mixing for a certain time, the fluorescence response signal corresponding to each concentration of target was recorded.

### 2.5. Procedures for SPE Enrichment Coupled with HPLC

The MIPs based on QD-grafted COFs (100 mg) were packed into 3 mL SPE cartridges. After the MIPs based on QD-grafted COFs cartridge was activated and conditioned with methanol and water (10 mL), respectively, a solution of FA in ethanol was loaded. After the retained analytes were eluted with acetic acid/methanol mixture (10:90, v/v, 5 mL), the eluent was dried under a gentle stream of nitrogen gas at 40 °C. The residue was dissolved in methanol and filtered through a 0.22 μm filter for subsequent HPLC analysis.

The HPLC chromatography analysis was achieved on an Inertsil ODS C18 (150 × 4.6 mm, 4.6 μm, Shimadzu, Kyoto, Japan). The mobile phase is a mixture solution of methanol/water (containing 0.5% HCOOH, 30:70, v/v) with a flow rate of 1.0 mL min^−1^. The detection wavelength for UV detector is 320 nm. All detected solutions were filtered through a 0.45 μm polytetrafluorethylene membrane before use.

## 3. Results

### 3.1. Synthesis and Characterization of the MIPs Based on QD-Grafted COFs

**Figure 1:**
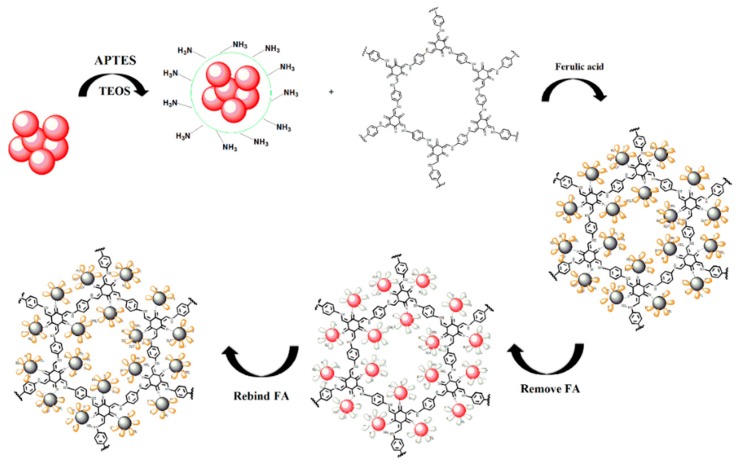
A schematic representation of the synthesis of molecularly imprinted polymers (MIPs) based on quantum dot-grafted covalent organic frameworks (QD-grafted COFs).

**Figure 2:**
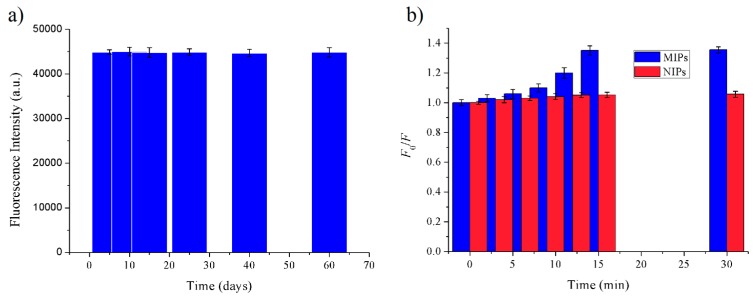
(**a**) The stability of MIPs based on QD-grafted COFs for ferulic acid (FA) molecules; (**b**) Adsorption Time of MIPs based on QD-grafted COFs for FA molecules.

The preparation of MIPs based on QD-grafted COFs used a one-pot surface-imprinting synthesis method (Figure 1). Here, the silane reagents of APTES and TEOS were used as the functional monomer and crosslinking agent, respectively. At the same time, the silane reagents were also used as QDs surface modification to provide –NH_2_ surface binding sites, inhibit effectively QDs photooxidation, improve the QDs fluorescent stability, and prevent the diffusion of the toxic heavy metal ions of QDs [28]. The QDs fluorescence intensity was quenched when the FA molecule was bound to the surface of the silica matrix by acid–base interactions; after the FA molecule was extracted from the matrix, the fluorescence intensity of the QDs recovered. The recognition sites were thus imprinted with FA molecules in the composites. The COFs were used as the support framework in the MIPs, and it reacted with APTES-modified QDs through a Schiff base reaction to form a stable structure. The MIPs based on QD-grafted COFs have a good chemical and optical stability because of the double stabilization of COFs and silica matrix. At room temperature, the MIPs based on QD-grafted COFs were stable for nearly 60 days (Figure 2a), indicating that the MIPs based on QD-grafted COFs possessed sufficient stability for FA analysis.

**Figure 3:**
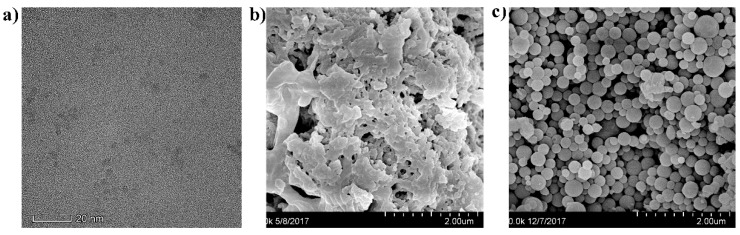
(**a**) Transmission electron microscope (TEM) image of QDs; (**b**) scanning electron microscope (SEM) image of COFs; (**c**) SEM image of MIPs based on QD-grafted COFs with a scale of 500 nm.

The transmission electron microscope (TEM) image of the QDs is shown in Figure 3a. It reveals that the QDs had spherical shapes and were well-dispersed in solution. The morphology of the as-prepared TPPA COFs is shown in Figure 3b. It revealed a TpPa series crystallized with a flowerlike morphology. The SEM morphology of the as-prepared MIPs based on QD-grafted COFs is shown in Figure 3c. It exhibits a uniform structure, highly spherical morphology, and rough surface, and the diameters were increased after coating with MIPs compared to the original QDs. The average sizes of QDs and MIPs based on QD-grafted COFs were measured by dynamic light scattering (DLS, Malvern Instruments Ltd., Malvern, UK) analysis as approximately 3 and 226 nm, respectively. This further revealed that the QDs were connected to the MIP system successfully.

**Figure 4:**
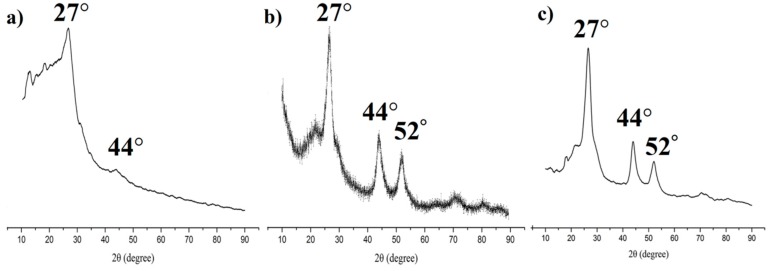
Powder X-ray diffraction spectrum of (**a**) COFs; (**b**) QDs; and (**c**) MIPs based on QD-grafted COFs.

The X-ray powder diffraction (PXRD) was used to obtain crystallinity of COFs (Figure 4a), QDs (Figure 4b), and the prepared MIPs (Figure 4c). These complexes showed sharp PXRD patterns, confirming their highly crystalline nature. The PXRDs of MIPs showed three main peaks which was a cubic structure with 2θ peaks at 27°, 44°, and 52°. These peak positions matched well with the PXRD patterns of QDs, and were similar with the PXRD patterns of COFs. This indicated that the MIPs coated on the COFs and QDs during the polymerization.

### 3.2. Fluorescent Properties of MIPs Based on QD-Grafted COFs

The photoluminescence quantum yields (QYs) were used to perform fluorescent properties of MIPs based on QD-grafted COFs. Rhodamine B (QY = 89%) was used as a reference. It was measured as 37% at room temperature. The QY was calculated using the equation below:

Φ_X_ = Φ_R_(*F*_X_/*F*_R_)(*A*_R_/*A*_X_)(η_R_/η_X_)

where Φ is the QY, *F* is the integral of fluorescence intensity, *A* is the absorbance at the excitation wavelength, and η is the refractive index of the solvent. X and R are the sample and reference, respectively.

### 3.3. Adsorption Properties

#### 3.3.1. Adsorption Time of MIPs Based on QD-Grafted COFs for FA Molecules

In order to investigate the binding performance of the MIPs and NIPs based on QD-grafted COFs, an equilibrium binding analysis was carried out using 15 mg kg^–1^ of FA. The adsorption time was investigated from 0 to 30 min, as shown in Figure 2b. The fluorescence intensity of QDs did not change with the increasing adsorption time until 15 min, above which the fluorescence intensity remained constant. Therefore, under the same concentration of FA, the optimal adsorption time of MIPs and NIPs based on QD-grafted COFs was 15 min. The change of fluorescence intensity from MIPs based on QD-grafted COFs was significantly higher than NIPs based on QD-grafted COFs. It indicated that the MIPs based on QD-grafted COFs formed more binding sites and cavities than NIPs based on QD-grafted COFs with the removal of template molecules, during the preparation process.

#### 3.3.2. Selectivity of the MIPs Based on QD-Grafted COFs

**Figure 5:**
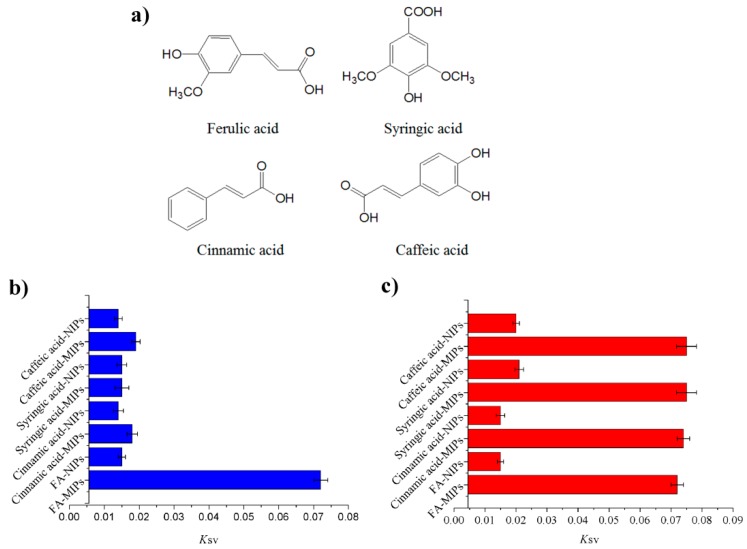
(**a**) The molecular structure of ferulic acid, cinnamic acid, syringic acid and caffeic acid; (**b**) The specificity determined using the Stern–Volmer plot for MIPs and non-imprinted polymers (NIPs) based on QD-grafted COFs for ferulic acid, cinnamic acid, syringic acid and caffeic acid; (**c**) The effects of 5 times concentration of competitive analogs on the binding of FA to MIPs and NIPs based on QD-grafted COFs.

The specificity of MIPs based on QD-grafted COFs was characterized with the structural and functional analogs of ferulic acid, cinnamic acid, syringic acid, and caffeic acid. Figure 5a showed the chemical structures of FA and its analogs. Figure 5b plots the Stern–Volmer slopes for FA and the analogs. The fluorescence quenching fractions of MIPs based on QD-grafted COFs induced by FA were significantly higher than those of its analogs, whereas differences quenching fractions between the analogs were not obvious. The fluorescence quenching fractions of NIPs based on QD-grafted COFs for FA were similar as its analogs, indicating that it did not form the specific binding sites in NIPs during the polymerization. In order to further investigate the effects of interfering substances on the binding of FA to MIPs and NIPs based on QD-grafted COFs, we used 5 times concentration of analogs to compare with the FA target. As shown in Figure 5c, the high concentration of analogs was not a significant effect on the results. Therefore, the MIPs based on QD-grafted COFs had poor selectivity for these analogs and the imprinting cavity left by removal of the template molecules enabled the analyte to access the receptor sites.

### 3.4. The Standard Curve and the Detection Limit of MIPs Based on QD-Grafted COFs

**Figure 6:**
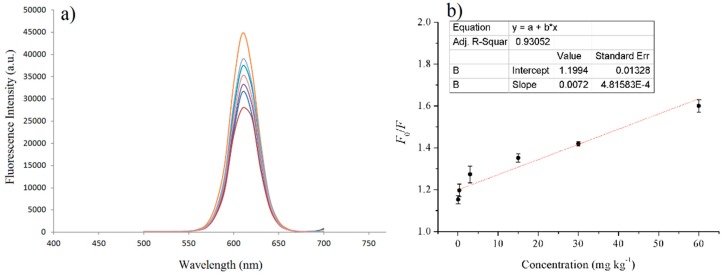
(**a**) Fluorescence spectra of MIPs based on QD-grafted COFs in the presence of increasing FA concentration; (**b**) Stern–Volmer plot of FA concentration and the fluorescence intensity of MIPs based on QD-grafted COFs.

For FA detection, the fluorescence spectra of the MIPs and NIPs based on QD-grafted COFs in the presence of FA were examined in ethanol at room temperature. In a typical experiment, a different amount of FA standard solutions was completely dissolved in ethanol and then 100 μL of the FA standard solution were mixed with 200 μL 1 mg mL^−1^ of MIPs based on QD-grafted COFs ethanol solution. The fluorescence quenching was determined after the MIPs based on QD-grafted COFs were added into the FA solution by shaking. After 15 min, the fluorescence spectra and fluorescence intensity of the different concentrations of FA standard solutions were recorded. The linear range of the standard curve was 0.03–60 mg kg^−1^ calculated by the fluorescence intensity and the change of FA concentration, and the R-square was 0.9305 (Figure 6). The detection limit was 5 μg kg^−1^, calculated by the FA concentration quenched three times the standard deviation of the blank signal to divide the slope of the standard curve. At the same time, it exhibited a good SPE adsorption ability, and the linear range of the SPE coupled with HPLC/MS was from 0.02 to 20 mg kg^−1^, with a detection limit of 3 μg kg^−1^.

### 3.5. FA Detection in Real Samples

The potential applications of grain by-products in alleviating or preventing chronic disease have rarely been investigated. Highland barley bran, wheat bran, corn silk, and vinasse were used in the study. The optimal extraction conditions for grain by-products were established as 6 mL 60% acetone heated to 60 °C for 40 min. The FA contents of the grain by-products were shown in Table 1. The determined results were not significantly different with fluorescence and solid phase extraction-high performance liquid chromatography/mass spectrometry (SPE-HPLC/MS) methods. In order to verify the accuracy and practicality of the developed fluorescence and SPE-HPLC/MS methods based on MIPs based on QD-grafted COFs, the recovery experiments were carried out by spiked with 50, 100, and 200 μg kg^−1^. Calculations of the recovery percentages revealed that 88–114% and 90–97% were recovered from the optosensing and SPE-HPLC/MS methods, respectively, indicating good detection results.

The fluorescence method in the study was low detection limit and high sensitivity for FA detection, with the advantages of its cost, simple, fast and efficient properties, and small reagent consumption. The SPE-HPLC/MS method using MIPs based on QD-grafted COFs was a good FA extraction and separation from complex sample matrixes, with the advantages of selectivity and accuracy. The two methods reported in the paper have their respective advantages for FA analysis.

## 4. Conclusions

In summary, we propose efficient and rapid fluorescence sensing and SPE-HPLC/MS methods for FA detection in real samples based on a molecularly imprinted polymer based on QD-grafted COFs. The nanomaterial was simple and facile to achieve FA quantitative detection by optosensing and SPE adsorption. It exhibited a high QY as 37% at room temperature. At room temperature, the MIPs based on QD-grafted COFs were stable for nearly 60 days, indicating that the MIPs based on QD-grafted COFs possessed sufficient stability for FA analysis. The high times concentration of analogs to perform the selective experiment showed a good selectivity of the nanomaterial was established through strong FA-APTES interaction. The linear range of fluorescence sensing and SPE-MPLC/MS methods were 0.03–60 mg kg^−1^ and 0.02–20 mg kg^−1^, and the detection limit was 5 µg kg^−1^, and 3 µg kg^−1^, respectively. The system was used for the efficient detection of ferulic acid in real samples with recovery values of 88–114% and 90–97% by fluorescence sensing and SPE-HPLC/MS methods, respectively, indicating a high selectivity and sensitivity.

## Figures and Tables

**Table 1:** The ferulic acid (FA) concentrations in the grain by-product extract.

The samples	Fluorescence method		SPE-HPLC/MS method	
	Content (mg kg^−1^)	RSD (%)	Content (mg kg^−1^)	RSD (%)
Highland barley bran	3.10 ± 0.12	3.87%	3.11 ± 0.09	2.89%
Wheat bran	1.64 ± 0.04	2.44%	1.63 ± 0.03	1.84%
Corn silk	2.33 ± 0.06	2.58%	2.33 ± 0.04	1.72%
Vinasse	1.67 ± 0.08	4.79%	1.66 ± 0.03	1.81%
